# Assessing anthelmintic resistance risk in the post-genomic era: a proof-of-concept study assessing the potential for widespread benzimidazole-resistant gastrointestinal nematodes in North American cattle and bison

**DOI:** 10.1017/S0031182020000426

**Published:** 2020-07

**Authors:** Russell W. Avramenko, Elizabeth M. Redman, Claire Windeyer, John S. Gilleard

**Affiliations:** 1 Faculty of Veterinary Medicine, Department of Comparative Biology and Experimental Medicine, University of Calgary, Calgary, Alberta, Canada; 2 Department of Production Animal Health, University of Calgary, Faculty of Veterinary Medicine

**Keywords:** Anthelmintic resistance, benzimidazole, *Cooperia*, deep-amplicon sequencing, drug resistance, *Haemonchus*, metabarcoding, nemabiome, *Ostertagia*

## Abstract

As genomic research continues to improve our understanding of the genetics of anthelmintic drug resistance, the revolution in DNA sequencing technologies will provide increasing opportunities for large-scale surveillance for the emergence of drug resistance. In most countries, parasite control in cattle and bison has mainly depended on pour-on macrocyclic lactone formulations resulting in widespread ivermectin resistance. Consequently, there is an increased interest in using benzimidazole drugs which have been used comparatively little in cattle and bison in recent years. This situation, together with our understanding of benzimidazole resistance genetics, provides a practical opportunity to use deep-amplicon sequencing to assess the risk of drug resistance emergence. In this paper, we use deep-amplicon sequencing to scan for those mutations in the isotype-1 *β*-tubulin gene previously associated with benzimidazole resistance in many trichostrongylid nematode species. We found that several of these mutations occur at low frequency in many cattle and bison parasite populations in North America, suggesting increased use of benzimidazole drugs in cattle has the potential to result in widespread emergence of resistance in multiple parasite species. This work illustrates a post-genomic approach to large-scale surveillance of early emergence of anthelmintic resistance in the field.

## Introduction

The detection of anthelmintic drug resistance in nematode parasites during the early phases of its emergence is problematic in both animals and humans. Methods for anthelmintic resistance detection that are based on phenotype, whether by *in vivo* drug efficacy or *in vitro* bioassay, lack sensitivity, with resistance typically only being detected once the parasite population comprises more than 25% drug-resistant individuals (Martin *et al.*, 1989; Coles *et al.*, 1992; Rendell, 2010; De Graef *et al.*, 2012). Consequently, by the time resistance is detected, effective mitigation strategies, such as the use of drug combinations and/or refugia management, are already severely comprised. Molecular diagnostic techniques that detect the causal mutations of resistance, or genetically linked markers, have the potential to allow resistance to be detected at an early stage and enable more effective evidence-based resistance management. Further, the identification of known drug resistance mutations at a low frequency in parasite populations that are phenotypically susceptible to anthelmintics may help assess the risk of resistance emergence under the influence of drug selection pressure. Current progress in parasite genomics is increasing our ability to elucidate the genetic basis of resistance in many parasite species (Doyle and Cotton, 2019). Combining the results of genomic research with new DNA sequencing and genotyping technologies will provide exciting opportunities for resistance surveillance and risk assessment. In this paper, we apply deep-amplicon sequencing, using the Illumina MiSeq short read sequencing platform, to detect low-frequency benzimidazole resistance-associated mutations in cattle and bison parasites as a proof-of-concept and example of a post-genomic approach to resistance surveillance.

The combined endo- and ectoparasite activity, convenient pour-on formulation and low cost of macrocyclic lactone drugs such as ivermectin have resulted in their widespread use for parasite control in cattle for decades. In contrast, although the benzimidazoles are highly effective against a large range of internal nematode parasites, their lack of ectoparasite activity and less convenient oral formulation has meant they have been less commonly used in cattle, particularly in North America. Macrocyclic lactone resistance is now common in gastrointestinal nematodes in North American cattle (Gasbarre *et al.*, 2009, 2015). Anecdotal evidence suggests that more cattle producers are beginning to consider using benzimidazole drugs for gastrointestinal nematode control and this trend is likely to continue as the awareness of ivermectin resistance grows. Many of the same gastrointestinal nematode species that occur in cattle also infect bison (Avramenko *et al.*, 2017, 2018). Due to the greater risk of clinical disease associated with gastrointestinal nematodes in bison than in cattle, although commercial bison herds still predominantly rely on pour-on macrocyclic lactones for control, there has been somewhat greater use of benzimidazole drugs in this host species (pers comm ref from Dr Roy Lewis, Merck Animal Health). Consistent with the relatively limited use, there has, as yet, only been a few cases of confirmed benzimidazole-resistant parasites in North American cattle (Gasbarre *et al.*, 2009; Chaudhry *et al.*, 2014). However, in countries where the benzimidazoles have been more heavily used in cattle, such as New Zealand, Australia and South America, resistance is becoming widespread in gastrointestinal nematodes of cattle (McKenna, 1991; Waghorn *et al.*, 2006; Cotter *et al.*, 2015; Ramos *et al.*, 2016).

We have a good understanding of the causal mutations most likely to be found in cattle trichostrongylid species from research in *Haemonchus contortus* and other phylogenetically related nematode parasites of sheep (Gilleard, 2013; Gilleard and Redman, 2016). Three non-synonymous single nucleotide polymorphisms (SNPs), F167Y (TTC > T**A**C), E198A (GAA > G**C**A) and F200Y (TTC > T**A**C), in the isotype-1 *β*-tubulin gene are associated with benzimidazole resistance in several sheep trichostrongylid gastrointestinal nematodes (Kwa *et al.*, 1994, 1995; Prichard, 2001; Ghisi *et al.*, 2007; Avramenko *et al.*, 2019). These amino acid codons (167, 198 and 200) have been shown to be important residues in the benzimidazole binding pocket and their effect on drug sensitivity has been functionally demonstrated (Aguayo-Ortiz *et al.*, 2013). The role of the F200Y (TTC > T**A**C) polymorphism in benzimidazole resistance has also been functionally confirmed in the free-living nematode *Caenorhabditis elegans* and *H. contortus*, a parasitic nematode of sheep (Kwa *et al.*, 1995). The F200Y (TTC > T**A**C) and F167Y (TTC > T**A**C) polymorphisms have shown to be under selection and/or associated with benzimidazole resistance in natural field populations for several parasitic nematodes of sheep including *H. contortus*, *Teladorsagia circumcincta*, *Trichostrongylus axei*, *Trichostrongylus colubriformis* and *Cooperia oncophora* among others (Grant and Mascord, 1996; Njue and Prichard, 2003; Gilleard, 2006; Avramenko *et al.*, 2019). For codon 198, a substitution of glutamic acid for alanine in *H. contortus* and glutamic acid for leucine substitution in *T. circumcincta* and *T. axei* have been also described in natural field populations (Redman *et al.*, 2015; Keegan *et al.*, 2017; Avramenko *et al.*, 2019). A substitution of glutamic acid for valine (E198V; GAA > **TT**A) in *T. circumcincta* and *T. axei* was also recently identified (Avramenko *et al.*, 2019).

We recently developed deep-amplicon sequencing methodology to screen for DNA sequence polymorphisms at codons 167, 198 and 200 of the isotype-1 *β*-tubulin gene in the major trichostrongylid nematode parasites of sheep (Avramenko *et al.*, 2019). Gastrointestinal nematodes in North American cattle and bison provide a practical situation in which a similar deep-amplicon sequencing approach can be used to screen drug resistance mutations either before, or at an early stage of emergence, to help assess the risk of phenotypic resistance emerging under the influence of drug selection pressure.

To date, no large-scale analysis of benzimidazole drug resistance mutations has been completed on cattle farms in either North or South America. In this paper, we apply our previously developed isotype-1 *β*-tubulin deep-amplicon sequencing assay to parasite populations isolated from beef cattle herds from Canada, the mid-southern USA and Sao Paulo State, Brazil and from both commercial and conservation bison herds from Western Canada. This paper represents the largest screen of benzimidazole resistance mutations in cattle and bison nematodes to date. It demonstrates a proof-of-concept of the detection of low-frequency drug resistance mutations in the early stages of emergence and suggests benzimidazole resistance has the potential to emerge quite quickly in cattle parasite populations if this drug class is applied to a greater extent in North American cattle and bison populations.

## Materials and methods

### Parasite material

#### Mid-southern US cattle herds

Samples used were originally described in Avramenko *et al.* (2017), and have been used in this paper with permission. Fecal samples were collected from 38 individual stocker cattle entering feedlot operations throughout 2014 in Oklahoma (22), Arkansas (3) and Nebraska (13). Each calf was from a different stocker cattle delivery from farms across the mid-southern USA, and thus likely represent animals derived from different farms. Collected fecal samples were cultured and stored as previously described.

#### Sao Paulo State, Brazilian cattle herds

Samples used were originally described in Avramenko *et al.* (2017), and have been used in this paper with permission. Fecal samples were collected from 26 farms, from calves 6–9 months of age, across Sao Paulo State in Brazil during August and September of 2015. Three individual fecal samples were collected per farm, and separately cultured before pooling all collected L3s into a single sample. Collected fecal samples were cultured as and stored previously described.

#### Canadian cattle herds

Samples used were originally described in Avramenko *et al.* (2017), and have been re-used in this paper with permission (Avramenko *et al.*, 2017). Fecal samples were originally collected from beef herds across Canada from June to December of 2012. Twenty individual fecal samples were collected per rectum or freshly voided on pasture, from groups of calves on pasture or entering feedlots. Fecal samples were collected with an approved Animal Use Protocol (Animal Care Committee, Study #AC13-0157, University of Calgary, Canada), which is in accordance with the principles outlined in the Guidelines of the Canadian Council on Animal Care. Exact details regarding sample collection can be found in Avramenko *et al.* (2017). Coprocultures were set up as described in Roberts and O'Sullivan (1950); however, vermiculite was used in place of sawdust, and samples were incubated at room temperature (~20°C). Third stage larvae (L3) were harvested from each individual fecal sample and subsequently pooled by herd. These pools of larvae were split into aliquots of ~1000–2000 when there were sufficient larvae. Larval aliquots were fixed in 70% ethanol and stored at −80°C until needed. Following quality assurance, 43 beef herd samples were included in the analysis; Alberta (19), Saskatchewan (7), Manitoba (2) and Ontario (15).

#### Canadian bison herds

Sampled bison herds were originally described in Avramenko *et al.* (2018) and have been reused in this paper with permission (Avramenko *et al.*, 2018). Exact information regarding sample collection and herd details can be found in Avramenko *et al.* (2018). Approximately 10–20 individual, freshly voided fecal pats were sampled and pooled from the pasture of each of the main production groups within a herd during farm visits, including cow–calf production groups (mature cows and bulls, replacement heifers and calves) and feeder production groups (yearling bison being fed for slaughter). Samples were collected between October 2014 and January 2015. Several of these production groups were managed by the same producer, making them part of the same overall herd. In addition, after quality assurance, 51 bison production group samples were included in this analysis: British Columbia (BC) (5), Alberta (16), Saskatchewan (15) and Manitoba (15). Further details regarding these samples can be found in Avramenko *et al.* (2018). In addition, freshly voided fecal samples were collected from individual bison from three conservation bison herds in Canadian national parks. These were Grasslands National Park (GNP) plains bison herd (*Bison bison bison*) (19 samples), Elk Island National Park (EINP) wood bison herd (*Bison bison athabascae*) (16 samples) and EINP plains bison herd (*B. bison bison*) (20 samples). Collected fecal samples were cultured and stored as previously described.

### Isotype-1 *β*-tubulin deep-amplicon sequencing

DNA preparations were made from the pooled larval samples as described in Avramenko *et al.* (2015). A fragment of the isotype-1 *β*-tubulin gene was amplified and sequenced from these pooled larval populations by deep-amplicon sequencing as previously described and validated in Avramenko *et al.* (2019). Sequencing was undertaken using the Illumina MiSeq, with the 2 × 250 bp v2 reagent kit (Illumina Inc., San Diego, CA, USA). Up to 384 samples were pooled in a single run and an average read depth of ~16 000 reads was obtained for each sample. Any samples, not providing a distinct first round PCR amplicon, as visualized on an agarose gel, or samples resulting in less than 2000 sequence reads were removed from the analysis as this is indicative of poor sample preparation.

### Bioinformatic analysis

Samples were analysed following the bioinformatic analysis pipeline outlined in Avramenko *et al.* (2019). In its current format, the deep-amplicon sequencing assay and subsequent bioinformatic pipeline are capable of detecting isotype-1 *β*-tubulin sequences from the following species: *C. oncophora*, *Cooperia punctata*, *Cooperia pectinata*, *Cooperia curticei*, *H. contortus*, *Haemonchus placei*, *T. circumcincta*, *Ostertagia ostertagi*, *Orloffia bisonis*, *T. axei*, *T. colubriformis* and *Trichostrongylus vitrinus*. For the purposes of this paper, the data were mined only for sequence variants at codons 167, 198 and 200 of the isotype-1 *β*-tubulin gene for each species.

### Nemabiome ITS-2 metabarcoding of populations

Nemabiome ITS-2 metabarcoding was previously carried out on all populations assessed in this paper. This provides the relative quantification of all nematode species present within mixed-species samples. Exact details regarding the method and the original results can be found in Avramenko *et al.* (2017) for the cattle samples from Canada, the USA and Brazil, while information pertaining to the Canadian bison samples can be found in Avramenko *et al.* (2018). The nemabiome barcoding technique is fully described in Avramenko *et al.* (2015) and at https://www.nemabiome.ca/index.html, and the results from these previous papers have been included with permission. Figures from both Avramenko *et al.* (2017, 2018) are reproduced in this paper under the terms of the Creative Commons Attribution 4.0 International License (http://creativecommons.org/licenses/by/4.0/). These data provide context for the species assessed for each sample, and are a reflection of the total percentage of the population a particular species represents.

## Results

Beef cattle herds from the mid-southern USA, Sao Paulo State, Brazil, Canada, and commercial and conservation bison herds from Western Canada were screened for the presence and frequency of non-synonymous SNPs at codons 167, 198 and 200 of the isotype-1 *β*-tubulin using a previously developed and validated deep-amplicon sequencing assay.

### Beef cattle from the mid-southern USA

**Fig. 1.**
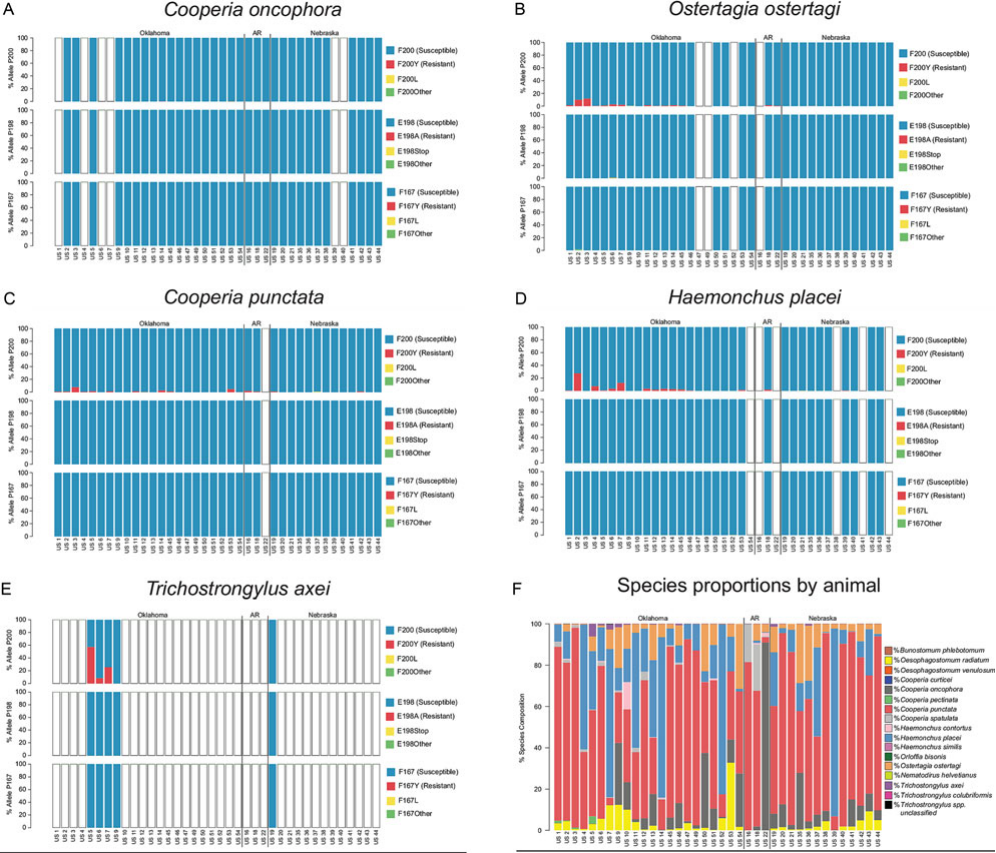
Benzimidazole resistance mutations in mid-southern US cattle herds. The allele frequency at codons 200, 198 and 167 of the *β*-tubulin isotype-1 gene is shown for *Cooperia oncophora* (A), *Ostertagia ostertagi* (B), *Cooperia punctata* (C), *Haemonchus placei* (D) and *Trichostrongylus axei* (E), derived from 38 individual stocker cattle entering feedlots in the mid-southern USA, as determined by deep-amplicon sequencing of a portion of the *β*-tubulin isotype-1 gene. Susceptible alleles are displayed in blue, while documented resistance alleles [F200Y (TTC > T**A**C), E198A (GAA > G**C**A) and F167Y (TTC > T**A**C)] are displayed in red. Other identified mutations at these codons are displayed in yellow and green. Blank bars indicate that the species was either not present in the sample, or there were too few sequences (<200) assigned to the species to assess the allele frequency. Panel F displays the relative proportion of each parasite species present in each herd as determined by nemabiome metabarcoding (ITS-2 rDNA deep-amplicon sequencing). These data were previously generated and published in Avramenko *et al.* (2017). AR, Arkansas.

A total of 38 individual stocker cattle entering feedlots in Oklahoma (22), Arkansas (3) and Nebraska (13) were assessed. Isotype-1 *β*-tubulin reads were identified in these samples, above the 200 reads per species threshold, for *C. oncophora*, *O. ostertagi*, *C. punctata*, *H. placei* and *T. axei*. The frequencies of non-synonymous mutations at codons 167, 198 and 200 were determined for these five species [Fig. 1, Table 1 (F200Y (TTC > T**A**C)), Table 2 (F167Y (TTC > T**A**C)), Supplementary Table S1 (F200L (TTC > TT**A**)) and S2 (F167L (TTC > TT**A**))]. The F200Y (TTC > T**A**C) SNP was found above the 0.1% species threshold in all five species, primarily in calves from Oklahoma; *C. oncophora* (five calves, frequency range 0.19–0.34), *O. ostertagi* (15 calves, frequency range 0.29–12.03%), *C. punctata* (16 calves, frequency range 0.18–7.75%), *H. placei* (15 calves, frequency range 0.57–27.45%) and *T. axei* (five calves, frequency range 8.49–57.43%) (Fig. 1 and Table 1). The F200Y mutation was also found in *H. contortus* in one US animal (US 10) at 1.7% [data not shown as *H. contortus* were only identified at >200 reads in two samples (US6 and US10)]. The F167Y (TTC > T**A**C) SNP was identified in just two of the five species at low frequency; *O. ostertagi* (four calves frequency range 0.13–0.38%) and *C. punctata* (two calves, range 0.23–0.3%) (Fig. 1, Table 2). The E198A (GAG > G**C**G) mutation was detected in *O. ostertagi* in one animal (US 6) at 0.51%, but was not detected in any other animal or species. The F167L and F200L SNPs were identified in *C. punctata*, *C. oncophora*, *O. ostertagi* and *H. contortus* in several animals at <1% frequency (Supplementary Tables S1 and S2).

**Table 1.** F200Y (TTC > TAC) resistance SNP frequencies by species and location

	Polymorphism	Species	# Herds/animals species detected	# Herds/animals SNP detected	Range (%)
Cattle: Canada (43 herds)	F200Y	*Cooperia oncophora*	43	0	N/A
		*Ostertagia ostertagi*	43	0	N/A
		*Cooperia punctata*	4	0	N/A
Cattle: USA (38 individual animals)	F200Y	*Cooperia oncophora*	32	5	0.19–0.34
		*Ostertagia ostertagi*	34	15	0.29–12.03
		*Cooperia punctata*	37	16	0.18–7.75
		*Haemonchus placei*	32	15	0.57–27.45
		*Trichostrongylus axei*	5	3	8.49–57.43
Cattle: Brazil (26 Herds)	F200Y	*Cooperia punctata*	25	1	0.25
		*Cooperia pectinata*	17	1	0.47
		*Haemonchus placei*	25	3	0.4–3.33
		*Haemonchus contortus*	3	3	48.78–90.22
		*Trichostrongylus axei*	8	0	N/A
Bison: Canada Commercial (51 production groups)	F200Y	*Cooperia oncophora*	50	0	N/A
		*Ostertagia ostertagi*	50	7	0.38–15.21
		*Orloffia bisonis*	8	1	0.58
		*Haemonchus placei*	12	0	N/A
		*Trichostrongylus axei*	10	0	N/A
Bison: Canada National Park (55 individual animals)	F200Y	*Cooperia oncophora*	46	0	N/A
		*Ostertagia ostertagi*	43	0	N/A
		*Orloffia bisonis*	19	0	N/A
		*Haemonchus placei*	0	0	N/A
		*Trichostrongylus axei*	28	0	N/A

**Table 2.** F167Y (TTC > TAC) resistance SNP frequencies by species and location

	Polymorphism	Species	# Herds/animals species detected	# Herds/animals SNP detected	Range (%)
Cattle: Canada (43 herds)	F167Y	*Cooperia oncophora*	43	0	N/A
		*Ostertagia ostertagi*	43	0	N/A
		*Cooperia punctata*	4	0	N/A
Cattle: USA (38 individual animals)	F167Y	*Cooperia oncophora*	32	0	N/A
		*Ostertagia ostertagi*	34	4	0.13–0.38
		*Cooperia punctata*	37	2	0.23–0.3
		*Haemonchus placei*	32	0	N/A
		*Trichostrongylus axei*	5	0	N/A
Cattle: Brazil (26 Herds)	F167Y	*Cooperia punctata*	25	1	0.13
		*Cooperia pectinata*	17	0	N/A
		*Haemonchus placei*	25	1	0.25
		*Haemonchus contortus*	3	3	2.22–40.58
		*Trichostrongylus axei*	8	0	N/A
Bison: Canada Commercial (51 production groups)	F167Y	*Cooperia oncophora*	50	0	N/A
		*Ostertagia ostertagi*	50	0	N/A
		*Orloffia bisonis*	8	0	N/A
		*Haemonchus placei*	12	0	N/A
		*Trichostrongylus axei*	10	0	N/A
Bison: Canada National Park (55 individual animals)	F167Y	*Cooperia oncophora*	46	0	N/A
		*Ostertagia ostertagi*	43	0	N/A
		*Orloffia bisonis*	19	0	N/A
		*Haemonchus placei*	0	0	N/A
		*Trichostrongylus axei*	28	0	N/A

### Beef cattle from Sao Paulo State, Brazil

**Fig. 2.**
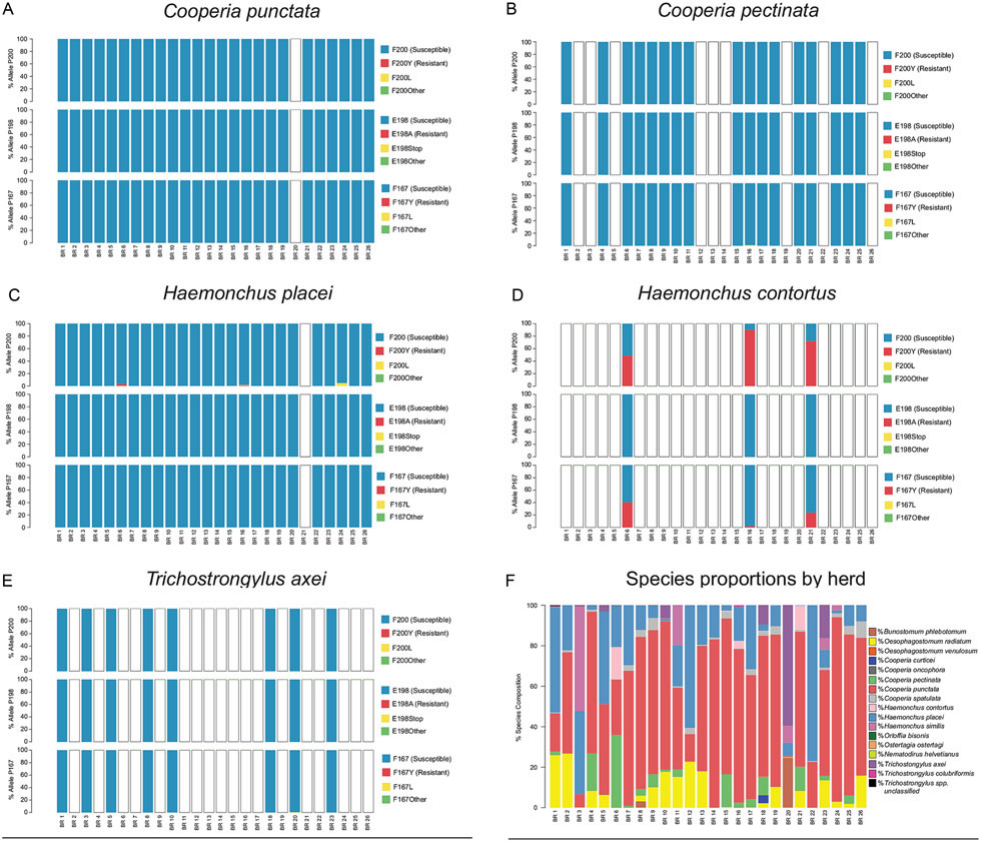
Benzimidazole resistance mutations in cattle herds from Sao Paulo State, Brazil. The allele frequency at codons 200, 198 and 167 of the *β*-tubulin isotype-1 gene is shown for *Cooperia punctata* (A), *Cooperia pectinata* (B), *Haemonchus placei* (C), *Haemonchus contortus* (D) and *Trichostrongylus axei* (E), derived from 26 herds in Sao Paulo State, Brazil, as determined by deep-amplicon sequencing of a portion of the *β*-tubulin isotype-1 gene. Susceptible alleles are displayed in blue, while documented resistance alleles [F200Y (TTC > T**A**C), E198A (GAA > G**C**A) and F167Y (TTC > T**A**C)] are displayed in red. Other identified mutations at these codons are displayed in yellow and green. Blank bars indicate that the species was either not present in the sample, or there were too few sequences (<200) assigned to the species to assess the allele frequency. Panel F displays the relative proportion of each parasite species present in each herd as determined by nemabiome metabarcoding (ITS-2 rDNA deep-amplicon sequencing). These data were previously generated and published in Avramenko *et al.* (2017).

Calves from a total of 26 herds from Sao Paulo State, Brazil were assessed; isotype-1 *β*-tubulin reads were identified in these samples, above the 200 reads per species threshold, for *C. punctata*, *C. pectinata*, *H. placei*, *H. contortus* and *T. axei.* The frequencies of non-synonymous mutations at codons 167, 198 and 200 were determined for these five species [Fig. 2 (F200Y (TTC > T**A**C)), Table 2 (F167Y (TTC > T**A**C)), Supplementary Table S1 (F200L (TTC > TT**A**)) and S2 (F167L (TTC > TT**A**))]. The F200Y (TTC > T**A**C) SNP was found above 0.1%, in *C. punctata* [one herd (BR6), 0.25%], *C. pectinata* [one herd (BR21), 0.47%], *H. placei* [three herds (BR6, BR15, BR16), range 0.4–3.33%] and *H. contortus* (three herds, range 48.78–90.22%). The F167Y (TTC > T**A**C) SNP was also found above 0.1% for *C. punctata* [one herd (BR21), 0.13%], *H. placei* [one herd (BR6), 0.25%] and *H. contortus* (three herds, range 2.22–40.58%). The F200L (TTC > TT**A**) SNP was detected in *C. punctata* (three herds, range 0.18–0.21%), *C. pectinata* (one herd, 0.14%) and *H. placei* (two herds, 0.51–4.68%). The F167L (TTC > TT**A**) SNP was detected in *C. pectinata*, *H. placei* and *T. colubriformis* in several herds <1%.

### Beef cattle from Canada

A total of 43 Canadian beef cattle herds were assessed comprising herds from Alberta (19), Saskatchewan (7), Manitoba (2) and Ontario (15). Consistent with the species composition of these herds, as previously determined by ITS-2 rDNA nemabiome sequencing of the same samples, isotype-1 *β*-tubulin reads mapping to just three species were identified; *C. oncophora*, *O. ostertagi* and *C. punctata* (Supplementary Fig. S1). The data for *C. punctata* were analysed for just those four herds for which there were at least 200 reads mapping to this parasite species (being the threshold that we have designated for reliable analysis). The frequencies of non-synonymous mutations at codons 167, 198 and 200 were determined [Supplementary Fig. S1, Table 1 (F200Y (TTC > T**A**C)), Table 2 (F167Y (TTC > T**A**C)), Supplementary Table S1 (F200L (TTC > TT**A**)) and S2 (F167L (TTC > TT**A**))]. At the detection frequency threshold of 0.1%, none of the F167Y (TTC > T**A**C), E198A (GAA > G**C**A) and F200Y (TTC > T**A**C) SNPs that have been previously associated with benzimidazole resistance in trichostrongylid nematodes were identified in any of these herds. The majority of worms had the susceptible genotype at each of the three codons. F167L (TTC > TT**A**) and F200L (TTC > TT**A**) SNPs, which have not been previously associated with benzimidazole resistance in previous studies, were identified at low frequencies in *C. oncophora* and *O. ostertagi* from several herds. An E198Stop (GAA > **T**AA) SNP was identified in *C. oncophora* in two herds (CAN14 and CAN19) at the frequencies of 2.07% and 2.78%, respectively.

### Commercial and conservation Bison herds from Western Canada

A total of 51 Canadian commercially farmed bison production groups from BC (5), Alberta (16), Saskatchewan (15) and Manitoba (15) were assessed. In addition, 55 individual animal samples from three Canadian national park conservation herds were assessed [GNP plains bison herd (19); EINP plains bison herd (16); EINP wood bison herd (20)]. Isotype-1 *β*-tubulin reads were identified in these samples, above the 200 reads per species threshold, for *C. oncophora*, *O. ostertagi*, *O. bisonis*, *H. placei* and *T. axei*. The frequencies of non-synonymous mutations at codons 167, 198 and 200 were determined for these five species [Supplementary Fig. S2 (F200Y (TTC > T**A**C)), Table 2 (F167Y (TTC > T**A**C)), Supplementary Table S1 (F200L (TTC > TT**A**)) and S2 (F167L (TTC > TT**A**))]. In the commercial bison herds, the F200Y (TTC > T**A**C) SNP was identified above 0.1% *O. ostertagi* (seven herds, range 0.38–15.21%) and *O. bisonis* (one herd, 0.58%), most of which were from Manitoba. The F167Y (TTC > T**A**C) SNP was not identified in any species from any herd. The F200L (TTC > TT**A**) SNP was identified in *C. oncophora* (three herds, range 0.25–0.68%), *O. ostertagi* (one herd, 2.76%) and *H. placei* (two herds, range 0.15–0.39%). The F167L (TTC > TT**A**) SNP was identified in *O. ostertagi* (five herds, range 0.31–0.98%), *H. placei* (two herds, range 0.11–0.29%) and *T. axei* (one herd, 0.11%).

In the conservation herds, neither the F200Y (TTC > T**A**C) nor F167Y (TTC > T**A**C) SNPs were identified in any species, in any animal. However, the F200L (TTC > TT**A**) SNP was identified in *C. oncophora* (five animals, range 0.12–0.39%), *O. ostertagi* (three animals, range 0.14–1.06%) and *T. axei* (four animals, range 0.2–40.55%). The F167L (TTC > TT**A**) SNP was identified in *C. oncophora* (two animals, range 0.91–2.4%), *O. ostertagi* (two animals, range 0.2–5.68%), *O. bisonis* (one animal, 0.18%) and *T. axei* (one animal, 0.18%).

## Discussion

### Benzimidazole resistance-associated SNPs were detected in multiple trichostrongylid nematode species in calves derived from multiple beef cattle herds in the mid/southern USA

There have been no large-scale studies specifically investigating benzimidazole drug resistance in cattle parasites in North America. A recent large study investigated the levels of anthelmintic resistance in 72 beef cattle herds across 19 US states (Gasbarre *et al.*, 2015). Evidence of macrocyclic lactone resistance was found on approximately 1/3 of surveyed farms, mainly for *Cooperia* spp. However, benzimidazole efficacy was only tested for five of these herds and there was a >90% reduction of egg counts in each of these cases. Benzimidazole resistance has only been clinically confirmed for *H. contortus* from parasites infecting cattle from the USA, and so benzimidazole resistance is not considered to be a major issue at present (Gasbarre *et al.*, 2009; Gasbarre, 2014). Nevertheless, the F200Y (TTC > T**A**C) SNP has been detected at low frequency in *H. placei* from Georgia, Florida and Arkansas, using a pyrosequence genotyping assay suggesting resistance is at the early stages of emergence in this parasite species (Chaudhry *et al.*, 2014). In this study, we have performed a much wider and deeper scan for benzimidazole resistance-associated SNPs in multiple parasitic nematode species in US beef cattle, compared to previous efforts. Parasites were harvested from individual stocker calves upon feedlot entry, each being derived from separate deliveries of stocker cattle entering feedlots, so as to broadly sample the parasites derived from 38 different stocker herds. We detected the F200Y (TTC > T**A**C) SNP in all of the six trichostrongylid nematode species identified (*C. oncophora*, *C. punctata*, *O. ostertagi*, *H. placei*, *H. contortus* and *T. axei*) (Fig. 1 and Tables 1 and 2). Of particular concern is the detection of the F200Y (TTC > T**A**C) SNP in *O. ostertagi*, generally considered to be the most pathogenic GI nematode of cattle, from as many as 15 out of 34 of the stocker cattle tested (Navarre, 2020). These were generally present at relatively low frequency but were as high as 12.03% in one calf. This suggests that benzimidazole resistance-conferring mutations, although still at the early stages of emergence, are already widespread in the parasite species in the mid-southern regions of the USA (particularly in Oklahoma).

The low frequency of benzimidazole resistance mutations that we have found in each parasite population means that resistance would not be phenotypically apparent and would not be detected by fecal egg count reduction test (FECRT) tests, particularly in mixed-species infections (Martin *et al.*, 1989). A similar situation is true for both *H. placei* and to a lesser extent *C. punctata*, both of which are also pathogenic nematodes capable of causing clinical disease and production loss. *Trichostrongylus axei* is one of the less common trichostrongylid nematode species in cattle and was only found in sufficiently high numbers to test for the presence of benzimidazole-resistant SNPs in just five stocker cattle tested. The F200Y (TTC > T**A**C) SNP was found in *T. axei* from four out of the five animals for which this parasite species was tested and was as high as 57.43% in one of these animals (US5). This suggests that benzimidazole resistance may be more advanced for this parasite species in the USA. However, since *T. axei* comprised only 6.1% of the parasite population in this sample, this level of benzimidazole resistance would still not have been detected using the standard FECRT without coproculture, which does not discriminate between different trichostrongylid nematode species. It will be interesting to investigate if this parasite species becomes more predominant under the influence of selection if benzimidazole use increases in US beef cattle. The F167Y (TTC > T**A**C) SNP was also identified in both *C. oncophora* (four animals) and *O. ostertagi* (two animals), though it was only found at a frequency of <1%.

Overall, these results demonstrate that benzimidazole resistance mutations are widespread, but generally still at low frequencies in the mid-southern USA in all the cattle nematode species we assessed. This suggests that, if benzimidazole drug use is increased in grazing cattle, widespread emergence of benzimidazole resistance is likely and could emerge quite quickly at multiple locations depending on the selection pressure applied. This suggests mitigation strategies, such as the use of drug combinations and refugia management, will be critical to prevent resistance emergence in this region. It will be important to undertake follow-up studies to track this emergence in US cattle in the coming years.

### Benzimidazole resistance-associated SNPs were rare and at very low frequency in cattle nematode species but at high frequency in *H. contortus* isolated from beef cattle from Sao Paulo State, Brazil

Although there have been relatively few published benzimidazole resistance studies in cattle in Brazil, the available evidence suggests that phenotypic resistance is higher than in the USA (Jaeger and Carvalho-Costa, 2017). Phenotypic benzimidazole resistance has been reported in several regions of Brazil (Brasil *et al.*, 2012; Ramos *et al.*, 2016, 2019; Jaeger and Carvalho-Costa, 2017). A study in Sao Paulo State published in 2007 reported five out of 25 beef cattle herds to have moderate to high levels of benzimidazole resistance (FECR of <90%), although the resistant parasite species was not determined and the presence of resistance mutations not assessed (Soutello *et al.*, 2007).

In our study of calves screened from 26 different herds from Sao Paulo State, the F200Y (TTC > T**A**C) SNP was only identified for a very small number of herds; *C. punctata* (one herd), *C. pectinata* (one herd) and *H. placei* (three herds) and was at very low frequency in each case (<4%) (Fig. 2, Table 1). The F167Y (TTC > T**A**C) mutation was also found in *C. punctata* and *H. placei* in just one herd for each and at very low frequency in each case (<0.5%) (Fig. 2, Table 2). Hence, the herd prevalence and frequencies of these two benzimidazole resistance-associated SNPs were very low in cattle parasite species in the region of Sao Paulo sampled. Indeed, the frequencies of these SNPs were lower than those found in the mid-southern US herds which is perhaps surprising. Interestingly, however, for *H. contortus*, found to be present in three different herds in this study, the F200Y (TTC > T**A**C) SNP was present at a very high frequency in each case (48.78–90.22%). The F167Y (TTC > T**A**C) SNP was also present at moderate to low frequencies in each of these cases (2.22–40.58%). *Haemonchus contortus* is primarily a sheep parasite, which can also infect cattle, and surveys of sheep have shown high levels of benzimidazole resistance throughout Brazil, including Sao Paulo State (Niciura *et al.*, 2012; Chagas *et al.*, 2016). Since co-grazing of sheep and cattle is relatively common in Brazil, much more so than in the USA or Canada, it is possible that many of the cases of phenotypic benzimidazole resistance reported in Brazilian cattle could be due to *H. contortus* infections. Interestingly, a recent study provides some support for this hypothesis: Molecular identification of the parasite species surviving benzimidazole treatments in seven different cattle herds in Rio Grande du Sul revealed the only parasite species surviving treatment was *H. contortus* (Ramos *et al.*, 2019). Further, cattle on these seven farms were all co-grazing with sheep. These results suggest a hypothesis in which the higher prevalence of phenotypic benzimidazole resistance in cattle parasites in Brazil compared to the USA could be due, at least in part, to cattle co-grazing with resistant parasite-infected sheep, rather than greater drug selection in the Brazilian cattle themselves. More work is required to explore this hypothesis further.

### Benzimidazole resistance-associated SNPs at codons 167, 198 and 200 were not detected in trichostrongylid nematode populations from 43 Canadian beef herds sampled in 2012

None of the previously described benzimidazole resistance-associated SNPs at codons 167, 198 and 200 of the isotype-1 *β*-tubulin gene were detected in the Canadian beef cattle herds by deep-amplicon sequencing using a sensitivity threshold of 0.1% frequency for each species in each population. Each of the samples used to generate DNA templates contain approximately 1000 L3 larvae in total, with *C. oncophora* and *O. ostertagi* comprising ~50% of the nematode populations each. Consequently, an average of ~500 L3 larvae, equating to ~1000 isotype-1 *β*-tubulin alleles, were screened for each species in each sample. Theoretically, this means that the lowest frequency that any allele can be present in any sample, corresponding to a single heterozygote, is 0.1%. Although we recognize that this could equate to lower than 0.1% frequency of sequence reads derived from a sample, due to the effect of amplification biases, we have used 0.1% as our operational detection threshold, to take a conservative approach. Nevertheless, we further explored the potential for *O. ostertagi* and *C. oncophora* being present below our 0.1% operational frequency threshold by determining the absolute number of each non-synonymous SNP at codons 167, 198 and 200 in the total dataset for each species when all samples were combined. For *O. ostertagi*, the F200Y (TTC > T**A**C) SNP was identified in 98 out of 276 852 sequences, and the F167Y (TTC > T**A**C) SNP in 23 out of 276 852 sequences; an overall prevalence of 0.035% and 0.008% across the 43 herds, respectively. For *C. oncophora*, the F200Y (TTC > T**A**C) SNP was identified in 212 out of 415 272 sequences, and the F167Y (TTC > T**A**C) SNP in 20 out of 415 272 sequences, giving them an overall frequency of 0.05% and 0.005% across the 43 herds, respectively. Consequently, it is possible that these benzimidazole resistance SNPs are present in the Canadian parasite populations at an extremely low frequency. However, at these very low frequencies, it is possible that these sequences were the result of contamination from other samples (which is unlikely as these mutations were not present at high frequency in any of the samples analysed in the whole study), or were more likely a result of PCR amplification or sequencing errors. More experimental work will need to be done to resolve this question.

The absence, or extremely low frequency, of the F200Y (TTC > T**A**C), E198A (GAA > G**C**A) and F167Y (TTC > T**A**C) resistance mutations in the Canadian cattle herds suggests that the emergence of benzimidazole resistance is less likely than in the mid-southern USA. This is probably due to even lower use of this drug class in the Canadian beef cattle cow–calf sector over the last few decades than in the USA (Hildreth and McKenzie, 2020; Navarre, 2020). It is important to note though that these samples were collected during 2012, and so it will be important to repeat similar studies over the next few years.

### Benzimidazole resistance-associated SNPs are present in *O. ostertagi* in parasite populations from Canadian bison herds

**Fig. 3.**
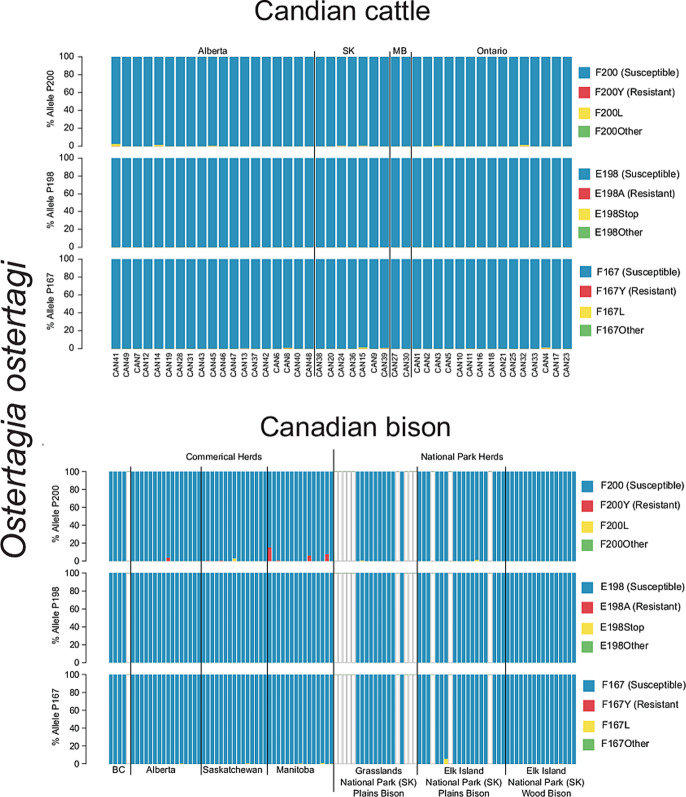
Benzimidazole resistance mutations in *Ostertagia ostertagi* from Canadian cattle and bison. The alleles frequency at codons 200, 198 and 167 of *β*-tubulin isotype-1 gene is shown for *Ostertagia ostertagi* derived from 43 commercial cattle herds (A). In addition, the allele frequencies of *O. ostertagi* derived from 51 commercial Canadian bison production groups, in addition to 55 individual bison samples from three National Parks, is also shown (B). Susceptible alleles are displayed in blue, while documented resistance alleles [F200Y (TTC > T**A**C), E198A (GAA > G**C**A) and F167Y (TTC > T**A**C)] are displayed in red. Other identified mutations at these codons are displayed in yellow and green. Blank bars indicate *O. ostertagi* was either not present in the sample, or there were too few sequences (<200) assigned to assess the allele frequency.

We detected the F200Y (TTC > T**A**C) benzimidazole resistance-associated SNP in *O. ostertagi* and *O. bisonis* in seven and one out of 50 commercial bison production groups, respectively. Although these were at relatively low frequency in most herds, the frequency was as high as 15.21% for *O. ostertagi* in one of the herds (Fig. 3). This contrasts with the result for the Canadian beef cattle herds in which the F200Y (TTC > T**A**C) SNP was not seen above the 0.1% threshold frequency for *O. ostertagi* in any of 43 herds assessed (Fig. 3). This result might be due to benzimidazoles being more commonly used in commercial bison herds due to the greater concern of clinical problems in bison than in cattle in Canada although we are not aware of any data to support this (pers. comm. Dr Roy Lewis). The finding of the F200Y (TTC > T**A**C) SNP present in *O. ostertagi*, derived from Canadian commercial bison herds, is of concern. This parasite is highly pathogenic in bison and the emergence of resistance to benzimidazoles would be a major problem for the industry both in terms of animal welfare and production. Furthermore, *O. ostertagi* is the most economically important and pathogenic parasitic nematode of cattle in temperate regions. Consequently, the presence of benzimidazole-resistant *O. ostertagi* in the Canadian grazing ecosystem presents a risk of transmission into cattle (Avramenko *et al.*, 2017, 2018). This risk will depend on the extent of pasture sharing/rotation between cattle and bison which needs to be carefully considered for the future management of anthelmintic resistance in both bison and cattle.

### Additional SNPs identified in codons 167, 198 and 200 of the isotype-1 *β*-tubulin gene and their potential for conferring benzimidazole resistance of trichostrongylid nematode populations

One of the inherent advantages of the deep-amplicon sequencing approach, compared with other diagnostic approaches such as real-time PCR and pyrosequence genotyping, is its ability to detect new unanticipated sequence polymorphisms. In this paper, we have confined the focus to the three codons of interest in the isotype-1 *β*-tubulin gene; 167, 198 and 200. Nevertheless, we identified several SNPs not previously reported in parasitic nematodes.

The F200L (TTC > TT**A**) and F167L (TTC > TT**A**) SNPs were identified at low frequencies across a variety of species in a number of herds from all locations surveyed (Fig. 1, Supplementary Figs S1–3). There is clear functional evidence that benzimidazole resistance is conferred by the F200Y (TTC > T**A**C) and F167Y (TTC > T**A**C) SNPs in nematodes and fungi (Roos *et al.*, 1990; Kwa *et al.*, 1993, 1994, 1995; Njue and Prichard, 2003; Gilleard, 2006; Kitchen *et al.*, 2019). In addition to these SNPs, the E198A (GAA > G**C**A), (E198 V GAA > G**T**A) and E198L (GAA > **TT**A) polymorphisms have clear functional evidence in fungi (Zou *et al.*, 2006; Banno *et al.*, 2008; Aguayo-Ortiz *et al.*, 2013; Liu *et al.*, 2014; Zhang *et al.*, 2016). Although there have been no such functional studies in nematodes for F200L (TTC > TT**A**) and F167L (TTC > TT**A**), the F167L (TTC > TT**A**) polymorphism has been implicated in benzimidazole resistance in fungi (Zou *et al.*, 2006; Msiska and Morton, 2009). Structural modelling studies suggest that the F167Y (TTC > T**A**C) and F200Y (TTC > T**A**C) SNPs reduce the binding affinity of benzimidazoles by increasing the polarity in the binding pocket while the E198A (GAA > G**C**A) SNP and E198L (GAA > **TT**A) polymorphism are predicted to disrupt the formation of a key H-bond interaction (Aguayo-Ortiz *et al.*, 2013). Hence it seems a reasonable hypothesis that the F200L (TTC > TT**A**) and F167L (TTC > TT**A**) SNPs could also affect benzimidazole binding affinity; however, further functional work in nematodes will be required to determine if this is the case.

A stop codon at codon 198 (GAA > **T**AA) of the isotype-1 *β*-tubulin gene was detected at a frequency of ~2% in two populations of *C. oncophora* from Canadian cattle (CAN14, CAN19). This mutation is predicted to result in a null allele and so a resistance phenotype. Null alleles have not been previously reported in the isotype-1 *β*-tubulin gene of trichostrongylid nematodes. This could be partly due to a bias in the approaches routinely used to screen for *β*-tubulin mutations which are unlikely to detect frameshift mutations or deletions. Alternatively, it could be due to a fitness cost to the loss of a functional copy of this gene, limiting the potential of null mutations to be selected to high frequency in the field. As with most previous studies, we have focused specifically on mutations previously shown to be associated with benzimidazole resistance. In future studies, it will be important to screen for additional mutations. Mining the data across the other codons in the amplified isotype-1 *β*-tubulin fragment in this study revealed premature stop codons in all species identified, from all regions sampled at codons 144, 153, 157, 158, 166, 172, 181, 183, 194, 208 (data not shown). In the majority of cases, these appeared at <1% frequency. The highest frequency was 18.5% at codon 183 in *C. oncophora* from farm CAN38. Such null alleles essentially remove the major drug target from the worm and so would be predicted to result in a resistance phenotype.

In conclusion, this study provides a proof-of-concept of the power of deep-amplicon sequencing to screen for anthelmintic resistance mutations and the value of the information generated. The rapid progress in sequencing technologies coupled with our increasing understanding of the genetic basis of anthelmintic resistance will make such approaches increasingly feasible for both animal and human helminths. In the latter case, such approaches are urgently required to assess the risk and monitor for anthelmintic resistance emergence in mass drug administration programmes.

## Supplementary material

For supplementary material accompanying this paper visit https://doi.org/10.1017/S0031182020000426. click here to view supplementary material
