# Supplementary material for: Assessing anthelmintic resistance risk in the post-genomic era: a proof-of-concept study assessing the potential for widespread benzimidazole-resistant gastrointestinal nematodes in North American cattle and bison
Source: Parasitology. 2020 Mar 6;147(8):897–906. doi: 10.1017/S0031182020000426 (PMC7391874; doi:10.1017/S0031182020000426)
Supplement: Supplementary file 1 [file S0031182020000426sup001.docx]

**
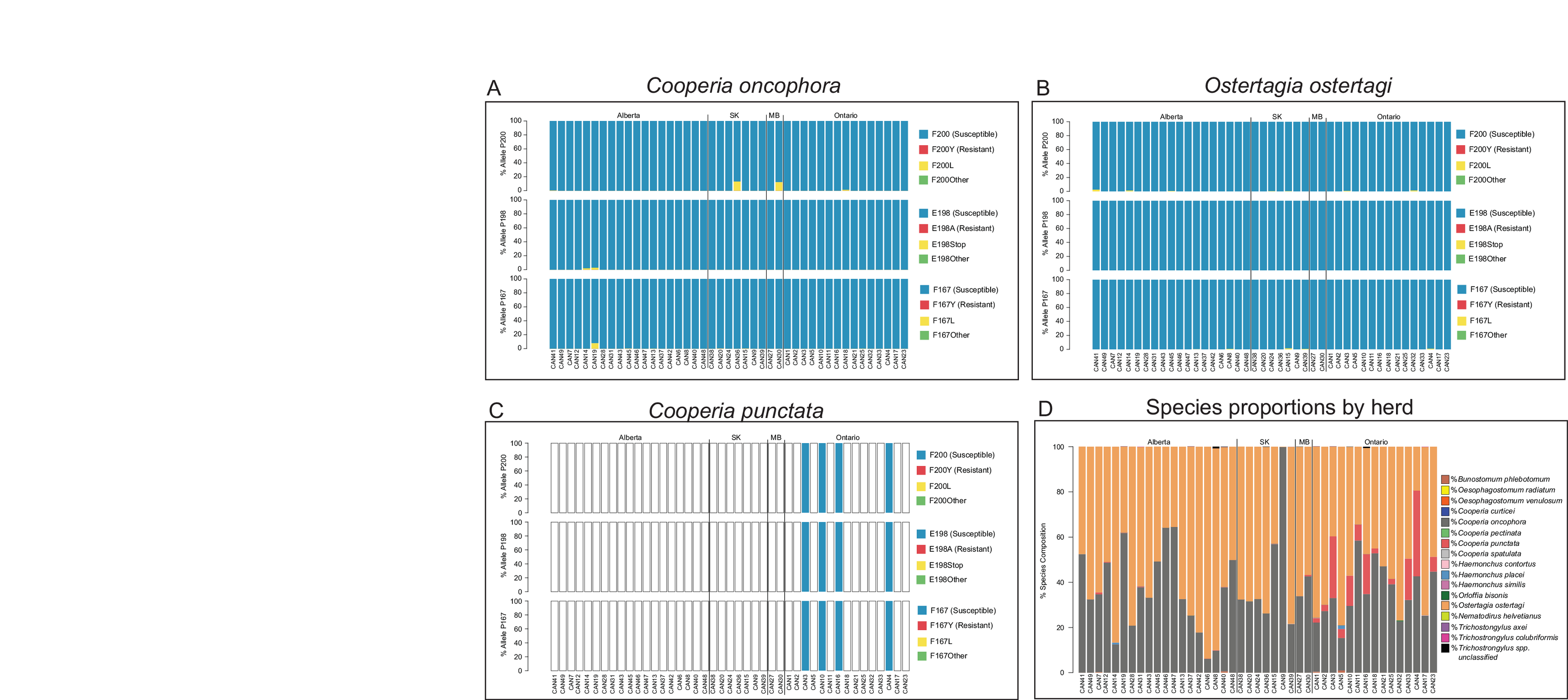
**

**Supplementary Fig. 1. Benzimidazole resistance mutations in Canadian cattle herds**

The allele frequency at codons 200, 198 and 167 of the β-tubulin isotype-1 gene is shown for *Cooperia oncophora* (A), *Ostertagia ostertagi* (B) and *Cooperia punctata* (C) derived from 43 Canadian cattle herds, as determined by deep-amplicon sequencing of a portion of the β-tubulin isotype-1 gene. Susceptible alleles are displayed in blue, while documented resistance alleles (F200Y (TTC>T**A**C), E198A (GAA>G**C**A) and F167Y (TTC>T**A**C)) are displayed in red. Other identified mutations at these codons are displayed in yellow and green. Blank bars indicate that the species was either not present in the sample, or there were too few sequences (<200) assigned to the species to assess the allele frequency. Panel D displays the relative proportion of each parasite species present in each herd as determined by nemabiome metabarcoding (ITS-2 rDNA deep amplicon sequencing). This data was previously generated and published in Avramenko *et al*, 2017. SK, Saskatchewan. MB, Manitoba


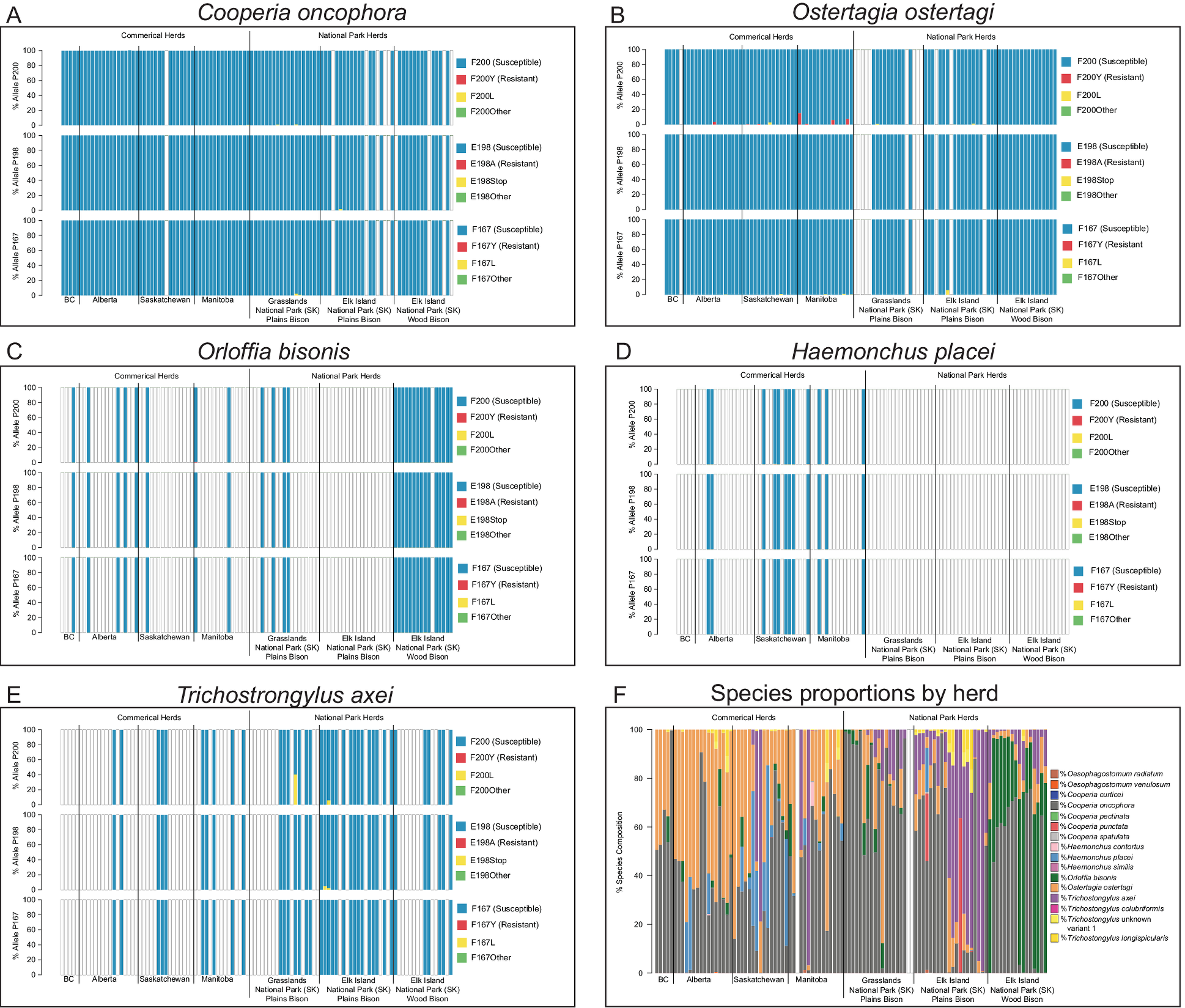


**Supplementary Fig. 2. Benzimidazole resistance mutations in Canadian bison from commercially farmed and national park conservation herds**

The allele frequency at codons 200, 198 and 167 of the β-tubulin isotype-1 gene is shown for *Cooperia oncophora* (A), *Ostertagia ostertagi* (B), *Orloffia bisonis* (C), *Haemonchus placei* (D) and *Trichostrongylus axei* (E), derived from 51 commercial Canadian bison production groups, in addition to 55 individual bison samples from 3 National Parks, as determined by deep-amplicon sequencing of a portion of the β-tubulin isotype-1 gene. Susceptible alleles are displayed in blue, while documented resistance alleles (F200Y (TTC>T**A**C), E198A (GAA>G**C**A) and F167Y (TTC>T**A**C)) are displayed in red. Other identified mutations at these codons are displayed in yellow and green. Blank bars indicate that the species was either not present in the sample, or there were too few sequences (<200) assigned to the species to assess the allele frequency. Panel F displays the relative proportion of each parasite species present in each herd as determined by nemabiome metabarcoding (ITS-2 rDNA deep amplicon sequencing). This data was previously generated and published in Avramenko *et al*, 2018. BC, British Columbia. SK, Saskatchewan.

**Supplementary Table 1. F200L (TTC>TTA) SNP frequencies by species and location**

|  | Polymorphism | Species | # Herds/animals species detected | # Herds/animals SNP detected | Range (%) |
| --- | --- | --- | --- | --- | --- |
| Cattle: Canada  (43 herds) | F200L | *Cooperia oncophora* | 43 | 4 | 0.47-13.14 |
|  |  | *Ostertagia ostertagi* | 43 | 7 | 0.56-2.6 |
|  |  | *Cooperia punctata* | 4 | 0 | N/A |
| Cattle: United States  (38 individual animals) | F200L | *Cooperia oncophora* | 32 | 0 | N/A |
|  |  | *Ostertagia ostertagi* | 34 | 3 | 0.12-0.48 |
|  |  | *Cooperia punctata* | 37 | 2 | 0.17-0.72 |
|  |  | *Haemonchus placei* | 32 | 2 | 0.19-0.25 |
|  |  | *Trichostrongylus axei* | 5 | 0 | N/A |
| Cattle: Brazil  (26 Herds) | F200L | *Cooperia punctata* | 25 | 3 | 0.18-0.21 |
|  |  | *Cooperia pectinata* | 17 | 1 | 0.14 |
|  |  | *Haemonchus placei* | 25 | 2 | 0.51-4.68 |
|  |  | *Haemonchus contortus* | 3 | 0 | N/A |
|  |  | *Trichostrongylus axei* | 8 | 0 | N/A |
| Bison: Canada  Commercial  (51 production groups) | F200L | *Cooperia oncophora* | 50 | 3 | 0.25-0.68 |
|  |  | *Ostertagia ostertagi* | 50 | 1 | 2.76 |
|  |  | *Orloffia bisonis* | 8 | 0 | N/A |
|  |  | *Haemonchus placei* | 12 | 2 | 0.15-0.39 |
|  |  | *Trichostrongylus axei* | 10 | 0 | N/A |
| Bison: Canada  National Park  (55 individual animals) | F200L | *Cooperia oncophora* | 46 | 5 | 0.12-0.39 |
|  |  | *Ostertagia ostertagi* | 43 | 3 | 0.14-1.06 |
|  |  | *Orloffia bisonis* | 19 | 0 | N/A |
|  |  | *Haemonchus placei* | 0 | 0 | N/A |
|  |  | *Trichostrongylus axei* | 28 | 4 | 0.2-40.55 |

**Supplementary Table 2. F167L (TTC>TTA) SNP frequencies by species and location**

|  | Polymorphism | Species | # Herds/animals species detected | # Herds/animals SNP detected | Range (%) |
| --- | --- | --- | --- | --- | --- |
| Cattle: Canada  (43 herds) | F167L | *Cooperia oncophora* | 43 | 6 | 0.11-7.76 |
|  |  | *Ostertagia ostertagi* | 43 | 5 | 0.27-1.42 |
|  |  | *Cooperia punctata* | 4 | 0 | N/A |
| Cattle: United States  (38 individual animals) | F167L | *Cooperia oncophora* | 32 | 2 | 0.1-0.2 |
|  |  | *Ostertagia ostertagi* | 34 | 0 | N/A |
|  |  | *Cooperia punctata* | 37 | 1 | 0.18 |
|  |  | *Haemonchus placei* | 32 | 1 | 0.22 |
|  |  | *Trichostrongylus axei* | 5 | 0 | N/A |
| Cattle: Brazil  (26 Herds) | F167L | *Cooperia punctata* | 25 | 0 | N/A |
|  |  | *Cooperia pectinata* | 17 | 1 | 1.07 |
|  |  | *Haemonchus placei* | 25 | 3 | 0.18-0.21 |
|  |  | *Haemonchus contortus* | 3 | 0 | N/A |
|  |  | *Trichostrongylus axei* | 8 | 2 | 0.17-0.52 |
| Bison: Canada  Commercial  (51 production groups) | F167L | *Cooperia oncophora* | 50 | 0 | N/A |
|  |  | *Ostertagia ostertagi* | 50 | 5 | 0.31-0.98 |
|  |  | *Orloffia bisonis* | 8 | 0 | N/A |
|  |  | *Haemonchus placei* | 12 | 2 | 0.11-0.29 |
|  |  | *Trichostrongylus axei* | 10 | 1 | 0.11 |
| Bison: Canada  National Park  (55 individual animals) | F167L | *Cooperia oncophora* | 46 | 2 | 0.91-2.4 |
|  |  | *Ostertagia ostertagi* | 43 | 2 | 0.2-5.68 |
|  |  | *Orloffia bisonis* | 19 | 1 | 0.18 |
|  |  | *Haemonchus placei* | 0 | 0 | N/A |
|  |  | *Trichostrongylus axei* | 28 | 1 | 0.18 |
